# The relationship between role clarity and compulsory citizenship behavior among junior nurses: evidence from network analysis and simulation-based network modeling

**DOI:** 10.3389/fpubh.2026.1861687

**Published:** 2026-07-08

**Authors:** Tingyao Nie, WeiKe Zeng, Hang Liu, Lu Lin, Miao Chen

**Affiliations:** 1 Department of Geriatrics, The First Hospital of Hunan University of Chinese Medicine, Hunan, China; 2 Contemporary Nurse Co., Ltd., Changsha, Hunan, China; 3 Nursing Teaching and Research Office, The First Affiliated Hospital of Zhengzhou University, Henan, China; 4 Xiangya School of Nursing, Central South University, Changsha, Hunan, China; 5 Department of Thoracic Surgery, The First Hospital of Hunan University of Chinese Medicine, Hunan, China

**Keywords:** Compulsory Citizenship Behavior, computer-simulated network intervention, junior nurses, network analysis, role clarity, workplace violence

## Abstract

**Background:**

Compulsory Citizenship Behavior (CCB) among junior nurses may undermine occupational wellbeing and workforce stability. Role clarity has been considered an important organizational resource, yet its item-level associations with CCB remain insufficiently understood. In addition, workplace violence (WPV) may shape the way in which role-related perceptions are associated with compulsory extra-role behaviors. This study aimed to explore the network associations between role clarity and CCB among junior nurses and to compare network structures between nurses with and without WPV exposure.

**Methods:**

A cross-sectional survey was conducted among junior nurses from hospitals in three provinces of China. Participants completed self-report questionnaires assessing role clarity, CCB, and WPV exposure. Network analysis was used to estimate item-level associations between role clarity and CCB. Bridge centrality indices were calculated to identify items that showed stronger statistical connections across constructs. Network comparison analyses were conducted between nurses with and without WPV exposure. Simulation-based prediction analysis was further performed to explore how changes in selected nodes might be statistically associated with changes in the overall network.

**Results:**

The estimated networks showed meaningful item-level associations between role clarity and CCB. Several items demonstrated relatively high expected influence or bridge expected influence, suggesting that they may occupy important positions in the statistical network. Network structures differed between nurses with and without WPV exposure, indicating that WPV exposure may be associated with different patterns of connections between role clarity and CCB. Simulation-based prediction suggested that changes in selected nodes may correspond to changes in predicted CCB levels within the estimated network model.

**Conclusion:**

This study provides preliminary evidence on the network associations between role clarity and CCB among junior nurses. The findings suggest that specific role-related and CCB-related items may serve as potential candidates for future organizational assessment or intervention development. However, because this study used a cross-sectional self-report design and simulation-based prediction rather than experimental intervention, causal interpretations should be avoided. Future longitudinal and intervention studies are needed to verify the temporal and causal mechanisms underlying these associations.

## Introduction

Nurses are widely recognized as the backbone of healthcare systems and play an essential role in achieving global health goals (1). They are central to universal health coverage, frontline patient care, primary care, and community health services (2). Nurses' professional competence and workforce stability are also critical for patient safety and the quality of clinical care, while investment in nurses' well-being can improve the safety, efficiency, and effectiveness of healthcare services (3). At a broader level, the nursing workforce contributes to health-system resilience during public health crises, epidemics, and natural disasters (4). Within the nursing workforce, junior nurses represent the new generation and future backbone of nursing services. Junior nurses currently account for 24.3% of China's registered nurses and are therefore important for maintaining the size and structure of the nursing workforce (5). This group is in a critical transition from academic training to clinical practice, during which they must adapt to clinical routines, professional responsibilities, and organizational expectations. However, high turnover among junior nurses has become a substantial challenge to nursing workforce stability (6). An attrition rate as high as 64.8% may result in substantial human resource loss and economic costs and may threaten the continuity, quality, and safety of nursing services (7). Therefore, understanding factors associated with junior nurses' occupational adaptation and workforce stability is important for nursing management and health-system sustainability.

In recent years, increasingly complex healthcare demands and rising organizational expectations have made nurses' professional environment more challenging. Under such conditions, nurses may experience pressure to engage in involuntary extra-role behaviors beyond their formal job scope, a phenomenon known as Compulsory Citizenship Behavior (CCB) (8). CCB refers to organizational citizenship behavior performed under perceived external pressure, mandatory requirements, or organizational normative pressure rather than voluntary willingness (9). In nursing settings, CCB may blur role boundaries, expand work responsibilities, and consume physical and psychological resources. Such resource depletion may be associated with reduced role clarity and increased role conflict (10). Existing studies have shown that CCB is associated with adverse occupational health outcomes, including increased work stress, burnout, and turnover intention among nurses (11). CCB has also been linked to psychological and behavioral variables, such as psychological contract-related perceptions and procrastination, which may further impair work performance and long-term wellbeing (12).

The association between role clarity and Compulsory Citizenship Behavior can be understood through an integrated theoretical framework combining Role Theory, the Job Demands–Resources (JD-R) model, Conservation of Resources (COR) theory, and Social Exchange Theory. According to Role Theory (13), employees rely on clear role expectations to understand the boundaries of their formal responsibilities. When role clarity is low, uncertainty regarding duties and performance expectations may increase, making employees more vulnerable to perceived pressure to engage in extra-role activities that are not formally required (13–15). For junior nurses who are still adapting to clinical environments and organizational norms, insufficient role clarity may blur the distinction between mandatory tasks and additional organizational demands, thereby increasing the likelihood of Compulsory Citizenship Behavior (16, 17). From the perspective of the JD-R model (18), role clarity can be regarded as an important job resource that helps employees cope with workplace demands. Adequate role clarity facilitates task prioritization, decision-making, and effective allocation of professional resources. Conversely, when this resource is insufficient, employees may experience greater strain and become more susceptible to organizational pressures that encourage involuntary extra-role behaviors (19–21). COR theory further suggests that individuals strive to acquire and preserve valuable resources (22). Continuous engagement in Compulsory Citizenship Behavior may consume emotional, cognitive, and physical resources, leading to resource depletion and increased occupational strain (17, 20). Therefore, role clarity may not only function as a protective resource but may also buffer the negative consequences associated with excessive extra-role demands (13). Social Exchange Theory provides an additional explanation for why employees may engage in Compulsory Citizenship Behavior (23). In organizational settings characterized by hierarchical relationships and performance evaluation systems, employees may perceive implicit obligations to reciprocate organizational support or satisfy supervisory expectations. When role expectations are ambiguous, nurses may perform additional duties beyond their formal responsibilities to gain approval, avoid criticism, or maintain favorable relationships with supervisors (24–26). Workplace violence (WPV) may further influence these processes (27, 28). As a significant occupational stressor, WPV can undermine employees' sense of safety, increase emotional exhaustion, and reduce available psychological resources (27). From both JD-R and COR perspectives, exposure to WPV may amplify the impact of insufficient role clarity by increasing vulnerability to external pressures and weakening employees' ability to resist involuntary organizational demands (27). Consequently, the associations between role clarity and Compulsory Citizenship Behavior may differ between nurses with and without WPV exposure.

Based on this integrated framework, the present study conceptualizes role clarity as a core organizational resource associated with Compulsory Citizenship Behavior and examines whether WPV exposure is related to differences in the network structure linking these constructs. Through network analysis and simulation-based prediction methods, this study aims to identify item-level associations, bridge nodes, and potential candidate targets for future organizational assessment and intervention research.

## Methods

### Study design and participants

This cross-sectional study employed convenience sampling to recruit junior nurses from 18 Grade A tertiary hospitals in Hunan, Guangdong, and Jiangxi provinces, China, between February 1 and April 30, 2025. These provinces were selected because they represent diverse healthcare contexts in central and southern China and include hospitals with varying organizational structures, staffing patterns, and clinical workloads. However, because convenience sampling rather than probability-based sampling was used, the sample should not be considered nationally representative of all junior nurses in China. Data were collected through the Wenjuanxing platform integrated into WeChat, which is widely used for online survey research in China.

The inclusion criteria were as follows: (1) possession of a valid nursing practice certificate issued in China; (2) having 1–3 years of clinical nursing experience; and (3) voluntary participation with informed consent.

The exclusion criteria were as follows: (1) nursing interns, nurses receiving advanced training, or rehired nurses; and (2) nurses who were absent from work because of illness, maternity leave, vacation, or other reasons during the study period.

To facilitate participant recruitment, the research team contacted nursing directors at each participating hospital and explained the study purpose, significance, and eligibility criteria. Nursing directors were invited to distribute the survey link to eligible nurses. To reduce potential self-selection bias, directors were asked to encourage participation from all eligible nurses regardless of their career development status, performance evaluations, or prior exposure to workplace stressors. Each mobile phone number was permitted to submit the questionnaire only once. Before completing the survey, all participants were required to read an electronic informed consent form describing the study purpose, voluntary nature of participation, confidentiality protections, and the right to withdraw at any time. No personally identifiable information was collected. After data screening, 1,084 valid questionnaires were included in the final analysis.

### Measures

#### Sociodemographic characteristics

Participants reported their gender, highest educational level, marital status, employment type, monthly income, workplace violence exposure, weekly overtime hours, and number of off-site training sessions during the previous year.

#### Role clarity scale

Role clarity was assessed using the Role Clarity Scale developed by Rizzo et al. (13) and translated into Chinese by Zhang Yanhong et al. (29). The scale consists of six items rated on a 7-point Likert scale ranging from 1 (strongly disagree) to 7 (strongly agree). Total scores range from 6 to 42, with higher scores indicating greater role clarity. The Cronbach's α coefficient in the present study was 0.816.

#### Compulsory citizenship behavior scale

Compulsory Citizenship Behavior (CCB) was assessed using the Compulsory Citizenship Behavior Scale developed by Vigoda-Gadot (30) and translated into Chinese by Lu Qiaoqiao (23). The scale consists of five items rated on a 5-point Likert scale ranging from 1 (strongly disagree) to 5 (strongly agree). Higher scores indicate greater levels of Compulsory Citizenship Behavior. The Cronbach's α coefficient in the present study was 0.809.

#### Workplace violence

Workplace violence (WPV) was assessed using a self-report screening item asking participants whether they had experienced any form of workplace violence during their nursing practice. WPV was defined as any incident in which nurses were abused, threatened, humiliated, harassed, or physically assaulted in circumstances related to their work. Examples included verbal abuse, threats, intimidation, physical violence, and other behaviors that could compromise nurses' psychological or physical safety. Participants reporting at least one experience of workplace violence were classified into the WPV group, whereas those reporting no workplace violence exposure were classified into the non-WPV group. This grouping strategy was used to examine whether the network associations between role clarity and Compulsory Citizenship Behavior differed according to workplace violence exposure.

### Data quality control

Several procedures were implemented to improve the quality of the online survey data.

First, each WeChat account and mobile phone number was restricted to one submission to prevent duplicate responses.

Second, mandatory-response settings were applied to minimize missing data.

Third, questionnaires completed in less than 5 min were excluded because pilot testing suggested that this duration was unlikely to allow participants to read and answer all questionnaire items carefully.

Fourth, questionnaires exhibiting highly repetitive response patterns across multiple scales were reviewed as potentially careless responses.

Because identical responses do not necessarily indicate invalid data, this criterion was applied cautiously and interpreted in conjunction with other indicators of careless responding. After data screening, 1,084 valid questionnaires remained for analysis.

### Common method bias

Because all study variables were measured using self-report questionnaires collected at a single time point, common method bias may have influenced the observed associations. To reduce this risk procedurally, participation was anonymous and voluntary, and respondents were informed that there were no right or wrong answers. Participants were also assured that their responses would be used solely for research purposes and would not affect their employment status or performance evaluations. In addition, Harman's single-factor test was conducted to evaluate common method variance. The first unrotated factor accounted for 35.15% of the total variance, which was below the commonly recommended threshold of 40%, suggesting that common method bias was unlikely to substantially influence the study findings.

### Statistical analysis

#### Data preprocessing

Except for descriptive statistics, which were performed using SPSS 29.0, other statistical analyses were conducted using R software (Version 4.5.2). Following the recommendations of Zhao et al. (31), item scores from the Role Clarity Scale and Compulsory Citizenship Behavior Scale were transformed using the nonparanormal transformation procedure implemented in the huge package to approximate multivariate normality before network estimation. For simulation-based network intervention analyses, item responses were dichotomized according to previously reported approaches (32). Specifically, Role Clarity items scored from 1 to 4 were coded as 0 and scores from 5 to 7 were coded as 1. For Compulsory Citizenship Behavior items, scores from 1 to 3 were coded as 0 and scores from 4 to 5 were coded as 1. Holm–Bonferroni correction was applied during network estimation analyses, whereas Benjamini–Hochberg false discovery rate correction was used when comparing simulation results.

#### Network estimation

Network structures were estimated using the extended Bayesian information criterion graphical least absolute shrinkage and selection operator (EBICglasso) method. This approach combines LASSO regularization with the EBIC criterion to obtain sparse and interpretable network structures. Expected influence (EI) and bridge expected influence (BEI) indices were calculated to identify central nodes and bridge nodes within the network. Bootstrap procedures were performed to evaluate the stability and accuracy of network estimates.

#### Simulation-based network intervention analysis

Simulation-based network intervention analyses were conducted using the NodeIdentifyR algorithm based on Ising network models. Because the Ising model requires binary indicators, all item responses were dichotomized before analysis. Although dichotomization enabled the application of Ising modeling, this procedure may have reduced information contained in the original Likert-scale responses and influenced network parameters. Therefore, findings from the simulation analyses should be interpreted as reflecting associations among dichotomized indicators rather than the full range of original responses. The simulation analyses were intended to identify statistically derived potential intervention targets and predicted changes in network activation. These findings should not be interpreted as evidence of actual intervention effectiveness or causal relationships.

### Ethical considerations

This study was approved by the Ethics Committee of the First Hospital of Hunan University of Chinese Medicine (Approval No. HN-LL-KYSB-2024-277). All participants provided electronic informed consent before participation. Participation was voluntary and anonymous, and all procedures complied with the principles of the Declaration of Helsinki. To ensure confidentiality, all data were stored on password-protected servers accessible only to the research team. Data analyses were conducted using fully anonymized datasets. Because this study did not involve clinical interventions or clinical trial procedures, clinical trial registration was not required.

## Results

### Topological overlap test

This study explores the association patterns between role clarity and Compulsory Citizenship Behavior through network analysis. The topological overlap test (threshold = 0.5) indicates good network stability, ensuring the reliability of the results. The network analysis identified 26 non-zero edges, with a network density of 47.27%, suggesting relatively close connections among the variables. Chi-square tests further revealed that, with the exception of item RC4 (“I have clear and specific goals and requirements for the work I perform,” *X*^2^ = 1.749, *p* = 0.096), the remaining items showed significant differences across subgroups with varying experiences of workplace violence, suggesting that workplace violence experience has a broad discriminatory effect on the relevant psychological constructs (see Table 1).

**Table 1:** Content of network nodes and their positive rates.

Scale	Item	Item content	Node name	Positive rate in the ising model (%)	
				Experience of workplace violence	No history of workplace violence
Compulsory citizenship behavior scale	1	Under pressure from my superiors, I have to make extra efforts to meet their demands	CCB1	23.17%	30.84%
	2	A culture of mandatory overtime within the department	CCB2	25.08%	33.92%
	3	My superiors always expect me to put in more effort at work	CCB3	45.81%	54.19%
	4	Even when I don't want to, I have to help my colleagues out of a sense of duty	CCB4	21.43%	31.94%
	5	Even if I don't want to, I have to volunteer to do some work that goes beyond my job responsibilities but helps my superiors	CCB5	26.98%	35.24%
Role clarity scale	1	I am very clear about the rights I have at work	RC1	61.43%	49.56%
	2	I know my job responsibilities	RC2	70.95%	66.30%
	3	I believe I have already allocated my personal work time reasonably	RC3	90.63%	93.17%
	4	I have clear and specific goals and requirements for the work I am responsible for	RC4	72.86%	68.94%
	5	I have a clear understanding of my expectations for the job	RC5	78.73%	74.89%
	6	I am unsure whether my work will be accepted by my superiors	RC6	34.13%	42.07%

### Ising networks and computer simulation of role clarity among junior nurses and compulsory citizenship behavior: selection of intervention targets

**Figure 1:**
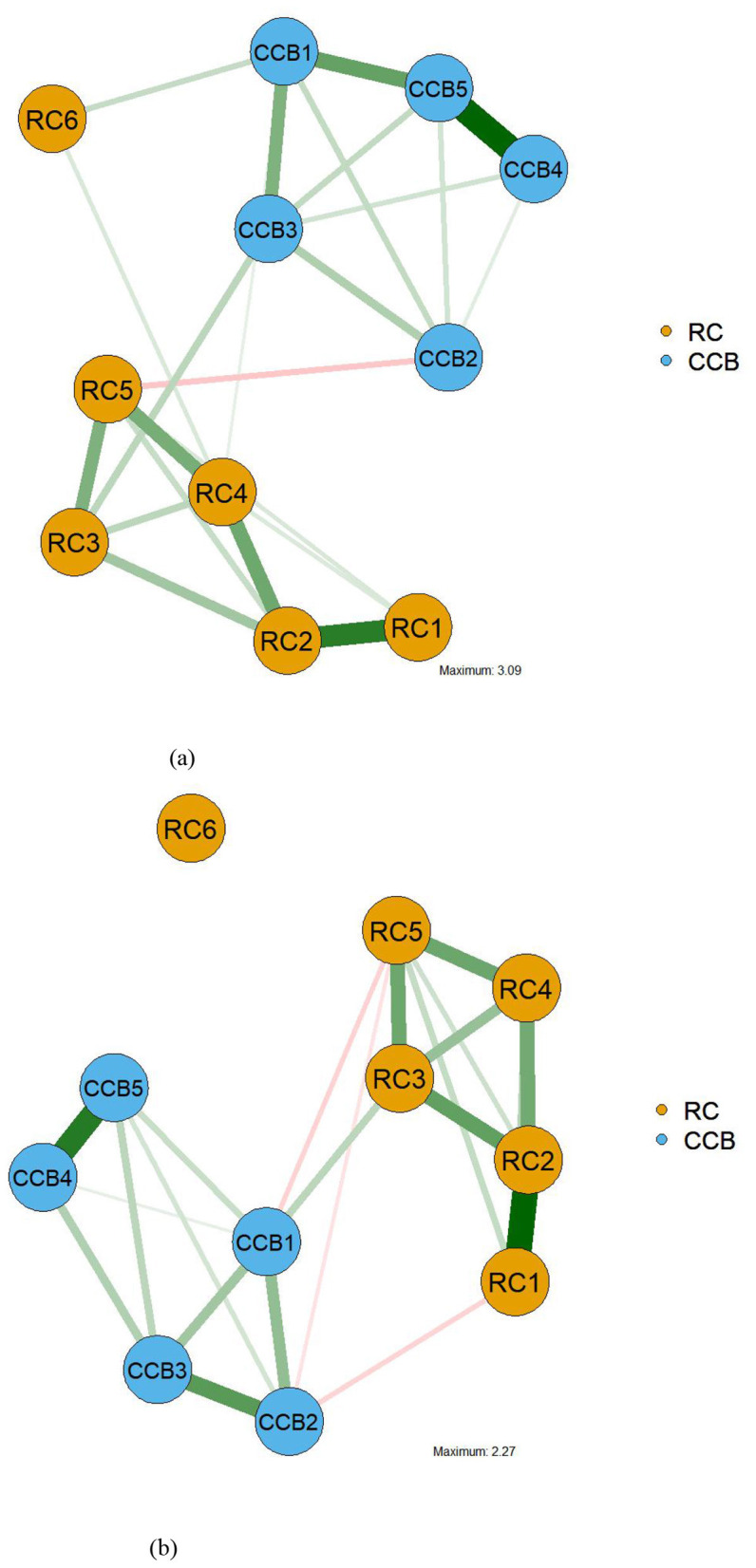
Ising-estimated interplay network of role clarity and CCB **(A)** Experienced workplace violence exposure group **(B)** Not experienced workplace violence exposure group.

**Figure 2:**
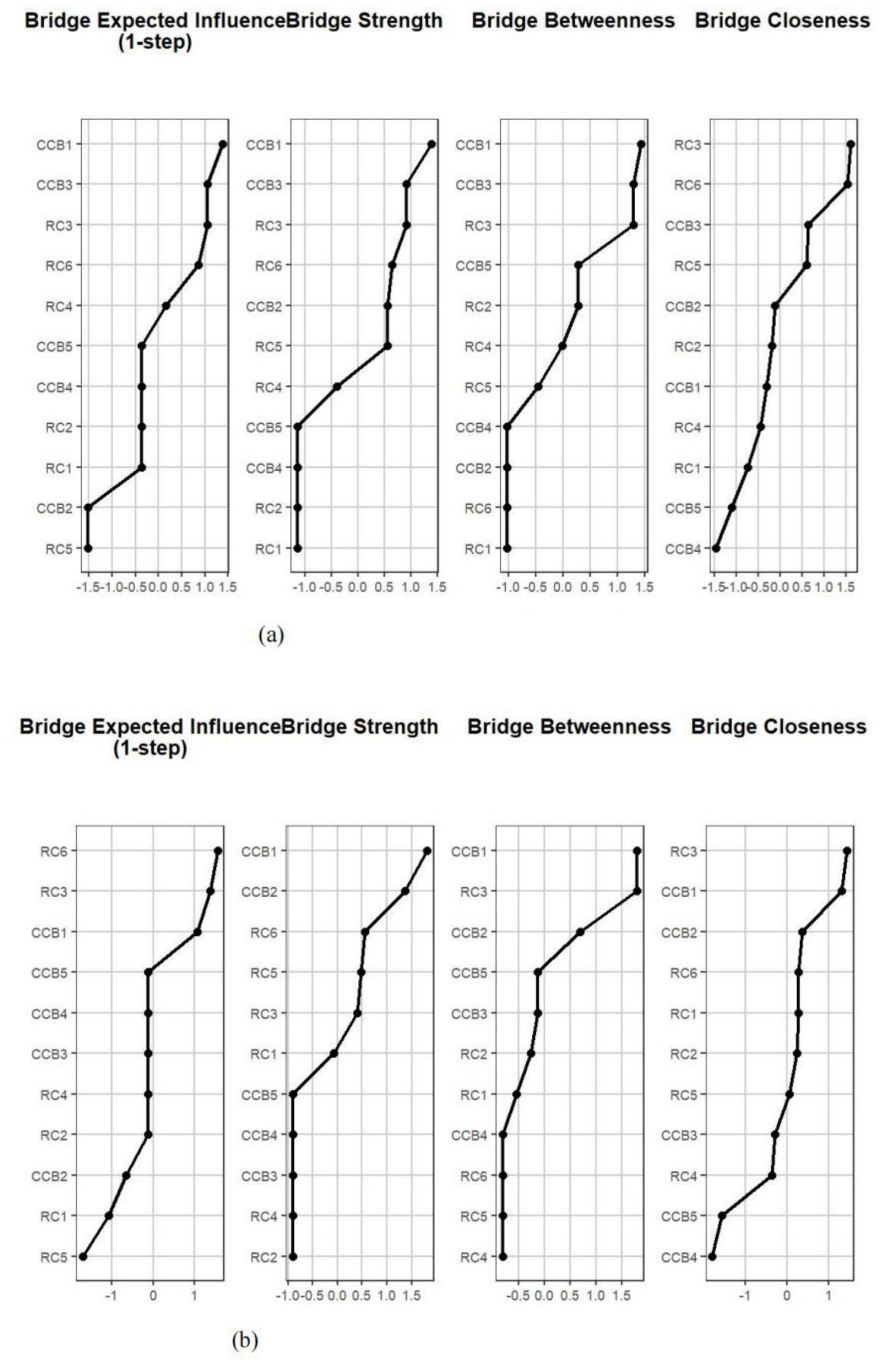
Bridging nodes in the Ising-estimated interplay network of role clarity and CCB. **(A)** Experience group of workplace **(B)** Not experience group of workplace.

Figures 1A, B present the analysis results of co-temporal networks for nurses who have experienced workplace violence and those who have not, respectively. In the co-temporal network clusters at the time points of experiencing and not experiencing workplace violence, the network structures exhibited stability, with good edge stability and expected strength stability. Specifically, the co-timely network at the time of experiencing workplace violence contained 23 non-zero edges, accounting for 41.82% of the total number of edges (55), with the strongest undirected non-zero edge being CCB4-CCB5 (*d* = 3.09). In contrast, the concurrent network at the time of no workplace violence experience contained 22 non-zero edges, accounting for 40.0% of the total edges (55), with the strongest undirected non-zero edge being RC1–RC2 (*d* = 2.267) (Figures 2A, B).

In the bridging network analysis, the key network bridge in the network at the time of experiencing workplace violence was CCB1 (bridge expected influence 1-step = 1.373). The key network bridge at the time of not experiencing workplace violence was RC6 (bridge expected influence 1-step = 1.556). Furthermore, a comparison of the networks at the time of experiencing workplace violence and the time of not experiencing workplace violence revealed that there was no significant difference in overall network strength between the two (experienced workplace violence = 4.408, not experienced workplace violence = 4.196, difference *S* = 0.213, *p* = 0.399). However, there was a significant difference in edge weights (*M* =0.236, *p* = 0.028), where M represents the maximum difference in edge weights between the two networks as computed by the Network Comparison Test (NCT). The centrality invariance test indicated that the strength of node RC5 differed significantly between the networks of those who had experienced workplace violence and those who had not (*p* = 0.021). The above results indicate that the networks at the same time points for those who have experienced workplace violence and those who have not are similar in overall structure, but exhibit structural heterogeneity in edge weights (network connections). It is therefore necessary to conduct a group-specific analysis based on whether or not workplace violence has been experienced.

### Validation of bridge nodes in EBICglasso networks of role clarity and compulsory citizenship behavior among junior nurses

**Figure 3:**
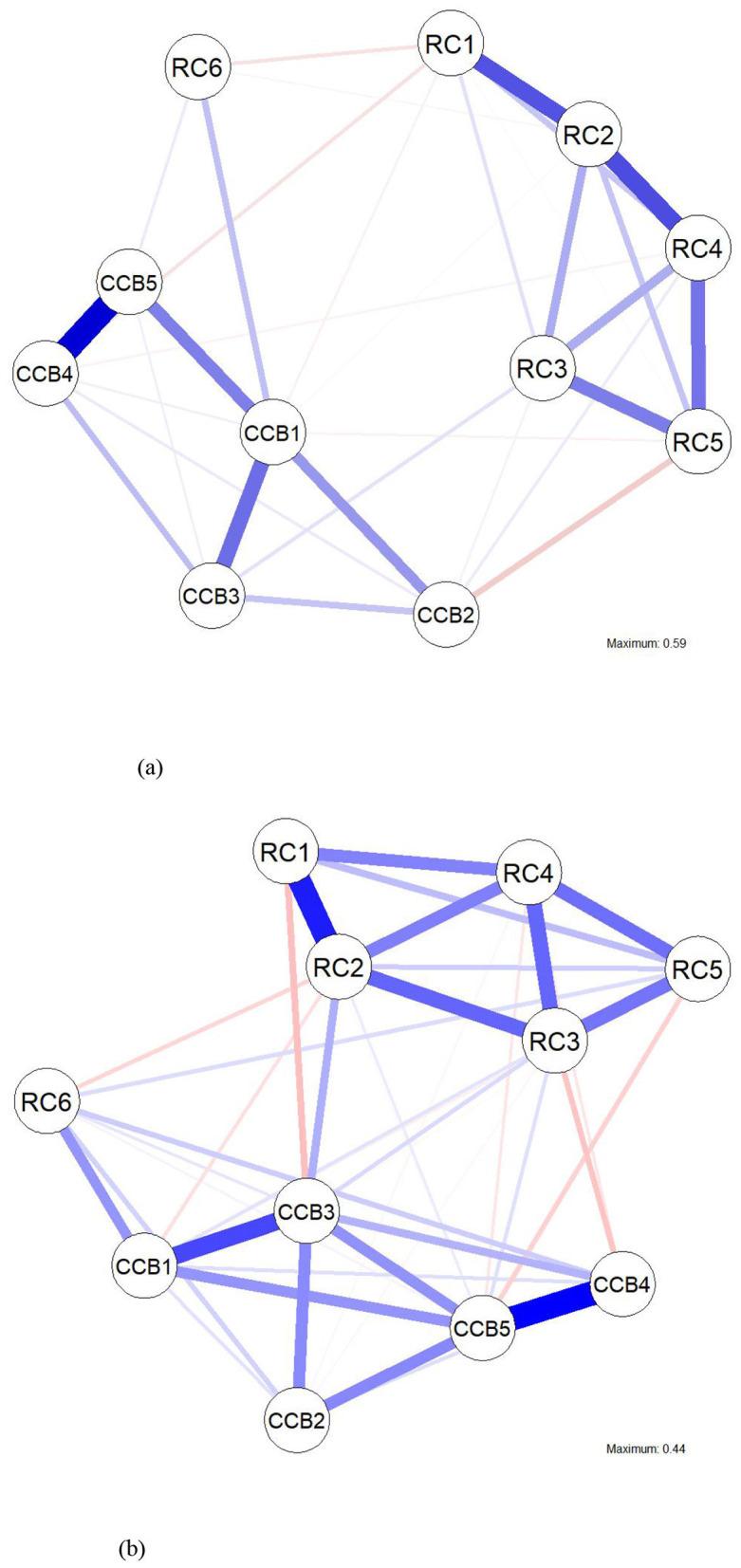
EBICglasso-estimated interplay network of role clarity and CCB **(A)** Experienced workplace violence exposure group **(B)** Not experienced workplace violence exposure group.

**Figure 4:**
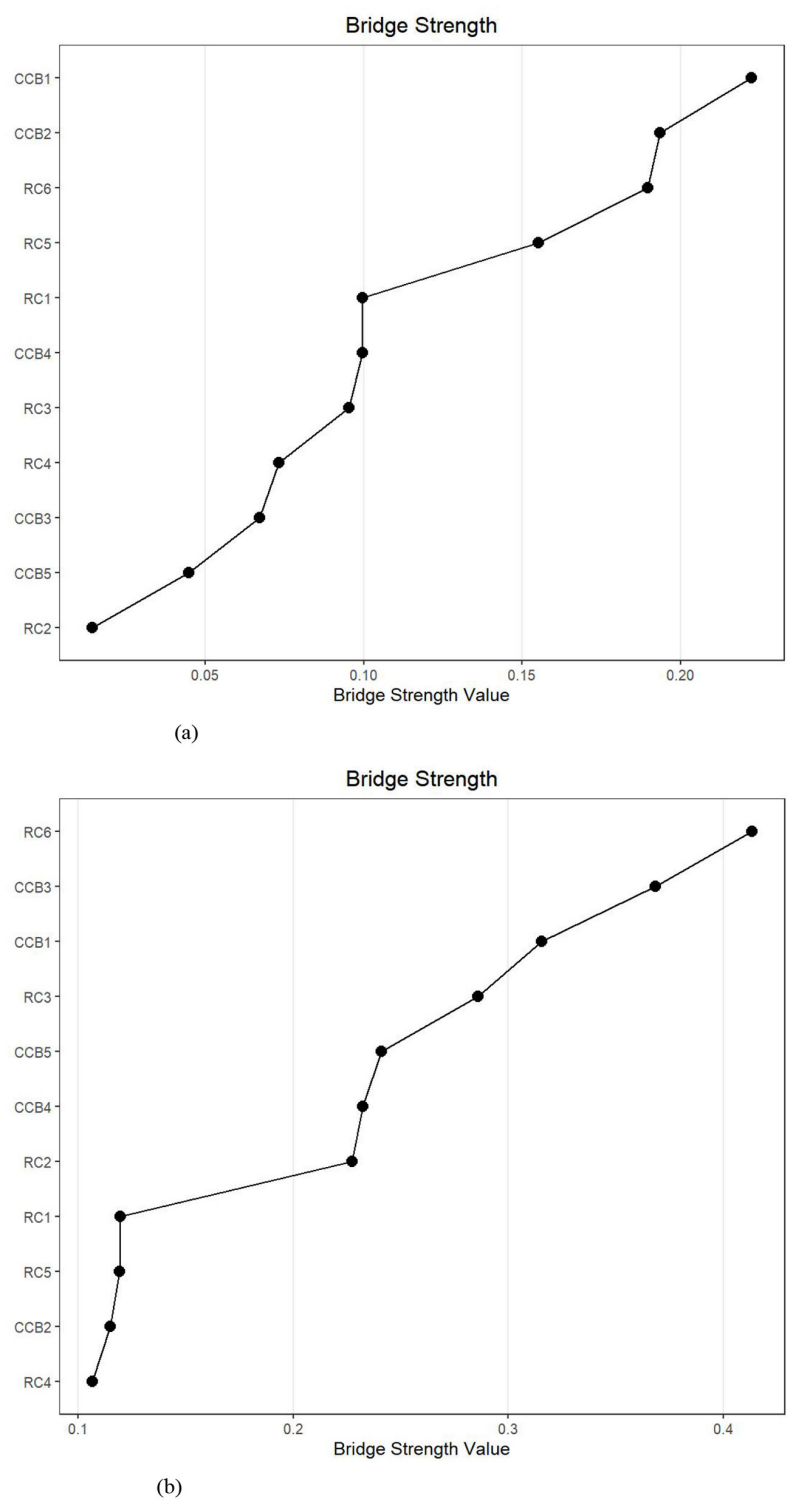
Bridging nodes in the EBICglasso-estimated interplay network of role clarity and CCB **(A)** Experienced workplace violence exposure group **(B)** Not experienced workplace violence exposure group.

Since the original Nurse Role Clarity Scale used a 7-point Likert scale and the Compulsory Citizenship Behavior Scale used a 5-point Likert scale, converting both scales to a 2-point Likert scale when constructing the Ising model may have altered the connections between bridging nodes. To verify whether the bridging nodes in the EBICglasso network and the Ising network were consistent across different subgroups, Following the approach in Wang (32), we fitted an EBICglasso model for role clarity and Compulsory Citizenship Behavior using the original scale scores (Figure 3) and recalculated the bridging expected influence index for each node in the EBICglasso network (Figure 4). The results showed that in the combined EBICglasso networks of role clarity and Compulsory Citizenship Behavior for nurses who had experienced workplace violence and those who had not, the nodes with the highest bridge strength, expected influence, bridge count, and bridge proximity were consistent with the Ising network results. Furthermore, to further examine the central stability of the item-level networks of role clarity and Compulsory Citizenship Behavior among different subgroups of nurses, the results indicated that the CS coefficients for bridging strength and bridging expected influence in the role clarity–Compulsory Citizenship Behavior network for nurses who had experienced workplace violence were both 0.751, whereas those for nurses who had not experienced workplace violence were both 0.672. This indicates that, regardless of whether in the Ising network or the EBICglasso network, the bridging nodes are stable, demonstrating good stability and generalizability, and are suitable for subsequent computer-simulated network interventions.

### Computer-simulated network intervention

**Figure 5:**
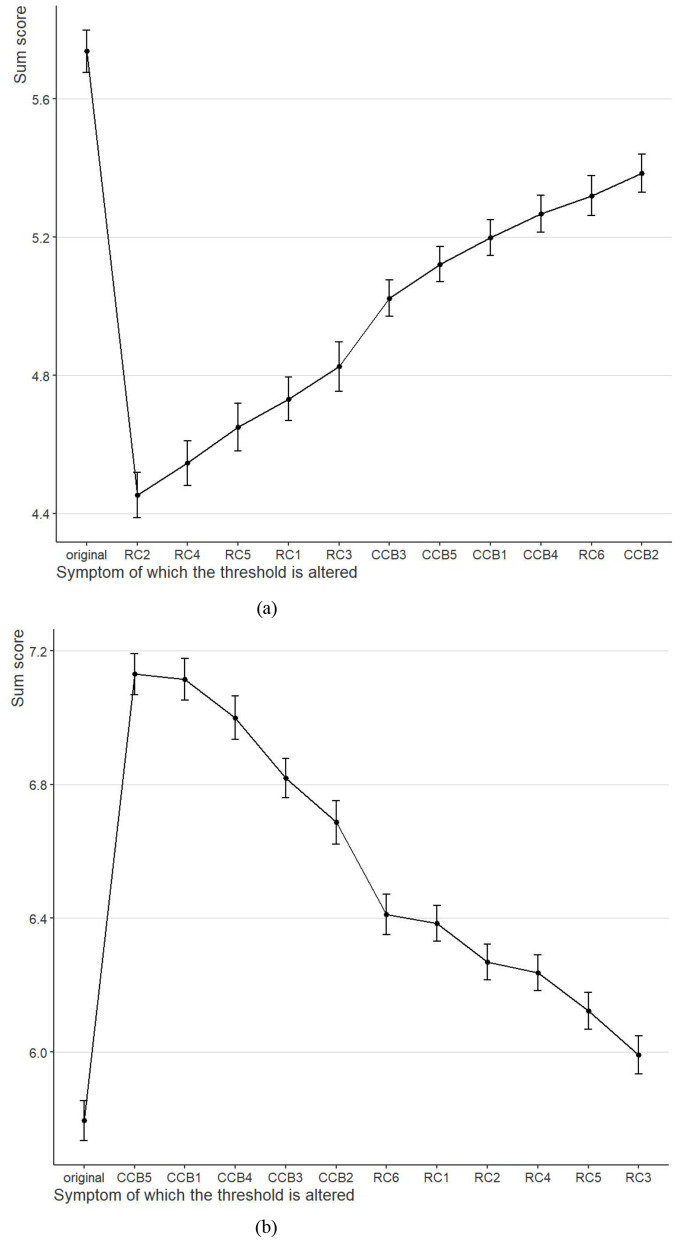
Computer-simulated network intervention in the workplace violence group. **(A)** Mitigation interventions **(B)** Enhanced intervention.

**Figure 6:**
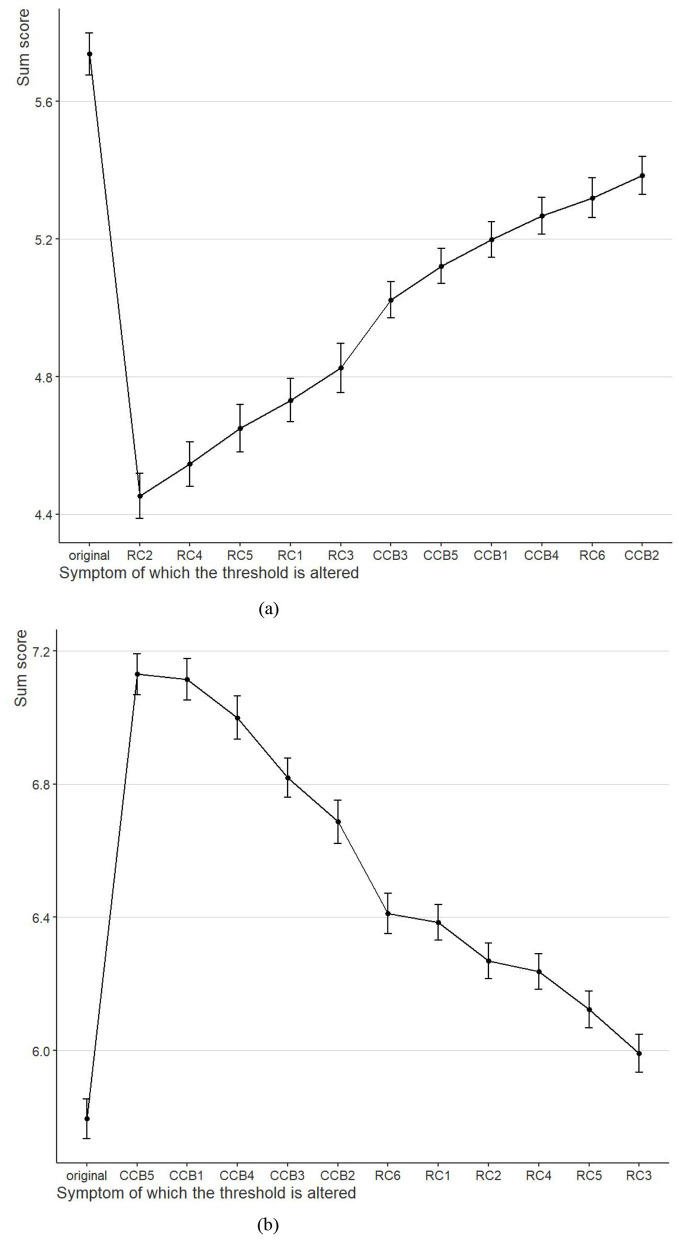
Computer-simulated network intervention in the non-workplace violence group **(A)** Mitigation interventions. **(B)** Enhanced intervention.

To assess which nodes in the joint network comprising role clarity and Compulsory Citizenship Behavior are key targets for exacerbating or alleviating overall network connectivity levels, we conducted dual-network computer simulation interventions on each node related to role clarity and Compulsory Citizenship Behavior in both the workplace violence-experienced network and the workplace violence-unexperienced network (using the NodeIdentifyR algorithm, NIRA), and compared the changes in the total scores for role clarity and Compulsory Citizenship Behavior under exacerbating and alleviating interventions, respectively. Based on the predicted effects of the mitigating and enhancing interventions, we ranked the results (as shown in Figures 5, 6). The simulation-based prediction analysis provided exploratory evidence regarding how hypothetical changes in selected nodes might be statistically associated with changes in the overall network structure. Within the combined network of role clarity and Compulsory Citizenship Behavior, several nodes emerged as potential candidate nodes showing relatively strong predicted associations with network activation. While some common patterns were observed across the workplace violence (WPV) and non-WPV subgroups, notable subgroup-specific differences were also identified. Specifically, regarding mitigation interventions, RC2 (“I know my job responsibilities”) was identified as the target requiring the highest priority across both subgroups, as it scored the lowest in the networks of both subgroups, indicating that enhancing RC2 would yield the greatest predicted mitigation effect. However, regarding aggravating interventions, the two subgroups exhibited distinct patterns: for the subgroup that had experienced workplace violence, CCB5 (“Even if I am unwilling, I am obligated to perform tasks beyond my job duties that assist my superiors”) scored the highest; whereas for the subgroup that had not experienced workplace violence, CCB4 (“My job requires me to help other colleagues complete their work”) scored the highest. This indicates that the vulnerable items most susceptible to being exploited to exacerbate negative behaviors differ across subgroup networks, and intervention efforts should focus on strengthening RC2 while remaining vigilant regarding CCB5 or CCB4 based on subgroup differences. We further compared whether there were significant differences between the original predicted total role clarity scores and the predicted total Compulsory Citizenship Behavior scores for each simulated intervention under exacerbation and mitigation scenarios (see Table 2).

**Table 2:** Changes in compulsory citizenship behavior and role clarity among junior nurses following computer-simulated interventions.

	Intervention pathway					
	Experienced workplace violence			No History of Workplace Violence (P)		
Intervention Type	Node	t	p	Node	t	p
Mitigation intervention	CCB1	1.657946e−39	—	CCB1	−8.851279	1.012593e−18
	CCB2	−13.210901	—	CCB2	−7.934317	2.344151e−15
	CCB3	−8.394394	5.324079e−17	CCB3	−18.546828	1.644915e−75
	CCB4	−17.447021	3.767784e−67	CCB4	−12.270843	2.307813e−34
	CCB5	−11.357776	1.030980e−29	CCB5	−14.836001	2.894754e−49
	RC1	−15.259090	5.771504e−52	RC1	−16.794818	1.903443e−62
	RC2	−22.497839	2.208312e−109	RC2	−25.626570	2.463067e−140
	RC3	−28.040808	1.156106e−166	RC3	−19.187776	1.367805e−80
	RC4	−19.075246	1.125811e−79	RC4	−24.079025	1.398231e−124
	RC5	−26.137487	1.046880e−145	RC5	−22.109511	8.630307e−106
	RC6	−23.126572	2.835603e−115	RC6	−8.076868	7.412793e−16
	CCB1	29.748625	2.119758e−186	CCB1	21.498599	2.927955e−100
	CCB2	19.720768	5.964598e−85	CCB2	20.594018	2.519878e−92
	CCB3	23.987126	1.139015e−123	CCB3	17.175901	3.467332e−65
Heavy intervention	CCB4	26.612771	8.652362e−151	CCB4	23.359821	1.485642e−117
	CCB5	30.377913	5.929675e−194	CCB5	22.240800	5.308366e−107
	RC1	14.374272	2.204357e−46	RC1	19.939980	9.243479e−87
	RC2	11.558420	1.054763e−30	RC2	16.590710	5.489611e−61
	RC3	4.651172	3.343008e−06	RC3	5.288003	1.262786e−07
	RC4	10.807039	4.521164e−27	RC4	14.163060	4.292286e−45
	RC5	7.932025	2.388899e−15	RC5	11.228369	4.425821e−29
	RC6	14.246671	1.312234e−45	RC6	10.041058	1.301289e−23

## Discussion

Based on a large sample, this study employed network analysis to explore the characteristics of the interaction network and its connecting bridges between role clarity and clusters of Compulsory Citizenship Behavior items among junior nurses. Furthermore, by integrating computer-simulated network intervention techniques from machine learning, the study conducted simulated interventions targeting the relationship between role clarity and Compulsory Citizenship Behavior across different subgroups with varying experiences of workplace violence. While providing theoretical guidance for frontline nursing managers, this study also offers a scientific basis for future work on the occupational health and psychopathology of junior nurses.

### Core bridging targets in the network of role clarity and compulsory citizenship behavior items among junior nurses

Network analysis results indicated that within the WPV-exposed subgroup network, CCB1 (“Under pressure from superiors, I must make extra efforts to meet their demands”) exhibited the highest bridge expected influence, suggesting that this item occupied a relatively important position in the network linking role clarity and Compulsory Citizenship Behavior (CCB) (9). The prominent bridging role of CCB1 suggests that perceptions of supervisory pressure and the obligation to exert additional effort may be closely associated with both role-related perceptions and CCB-related experiences in nurses who have experienced workplace violence (33). One possible interpretation is that WPV-exposed nurses may be more sensitive to hierarchical pressures and workplace interpersonal dynamics. Under such circumstances, requests from supervisors for additional work may be perceived as more difficult to decline, potentially contributing to stronger associations between role-related uncertainty and compulsory extra-role behaviors (34). However, these findings reflect statistical associations within the estimated network and should not be interpreted as evidence of causal mechanisms. From a practical perspective, nursing managers may consider monitoring perceptions of supervisory pressure and ensuring that requests for additional work are clearly communicated, voluntary when appropriate, and supported by transparent organizational procedures (28, 35). Organizations may also benefit from providing safe reporting channels and reinforcing policies that discourage coercive management practices.

In the subgroup without workplace violence exposure, RC6 (“I am unsure whether my individual work will be accepted by my supervisor”) exhibited the highest bridge expected influence, suggesting that concerns regarding performance recognition may occupy a relatively important position in the network structure. This finding may indicate that uncertainty regarding supervisory acceptance is more closely associated with both role clarity and CCB-related experiences among nurses who have not experienced workplace violence (36). One possible explanation is that insufficient clarity regarding performance expectations and feedback processes may be associated with increased uncertainty about work outcomes. Under such circumstances, some nurses may perceive additional efforts beyond formal job requirements as a way to gain recognition or demonstrate competence (30). Nevertheless, the present findings do not establish causal pathways and should be interpreted as exploratory associations derived from a cross-sectional network model. Based on these findings, managers may consider strengthening performance feedback systems, clarifying evaluation criteria, and providing timely communication regarding work expectations. Such measures may help reduce uncertainty surrounding role expectations and performance recognition. In addition, recognition systems may benefit from emphasizing performance within formal job responsibilities rather than rewarding excessive extra-role efforts (10, 37).

Overall, CCB1 and RC6 may represent potentially important indicators within the estimated network structures of the two subgroups. Future longitudinal and intervention studies are needed to determine whether these items play a meaningful role in the development or maintenance of CCB-related experiences. Promoting clear role boundaries, transparent communication, and supportive organizational practices may be beneficial areas for future organizational management and intervention research.

### Simulation-based prediction of candidate nodes in the network linking role clarity and compulsory citizenship behavior

The simulation-based prediction analysis provided exploratory information about how hypothetical changes in selected nodes might be statistically associated with predicted changes in Compulsory Citizenship Behavior within the estimated network model (8, 9). Across subgroups, RC2 (“I know my job responsibilities”) showed a relatively strong association with predicted decreases in CCB levels under the hypothetical improvement scenario. This finding suggests that clear understanding of job responsibilities may be an important candidate area for future organizational assessment and intervention development. From the perspective of Role Theory, clear job responsibilities may help junior nurses distinguish between formal role obligations and non-mandatory extra-role expectations (13). In early career stages, nurses are still adapting to clinical routines, professional norms, and hierarchical organizational structures (12). When job responsibilities are unclear, junior nurses may be more likely to perceive additional work demands as unavoidable obligations (38, 39). Therefore, improving clarity regarding job boundaries, responsibilities, and reporting lines may help reduce ambiguity in daily nursing work. However, the present findings should be interpreted cautiously. The simulation-based prediction analysis was derived from a cross-sectional statistical network model and did not involve actual intervention implementation. Therefore, the results cannot demonstrate that improving RC2 would directly reduce CCB in real-world clinical settings. Instead, RC2 should be regarded as a candidate node that may warrant further examination in longitudinal or intervention studies.

Subgroup-specific patterns were also observed. In the WPV-exposed subgroup, CCB5 showed a relatively high predicted increase under the hypothetical worsening scenario, suggesting that forced assistance toward superiors may represent a vulnerable indicator among nurses exposed to workplace violence. Nurses who have experienced WPV may be more sensitive to power imbalance, supervisory pressure, and perceived organizational insecurity (28, 40). In such contexts, requests for extra-role assistance from superiors may be more likely to be perceived as coercive or difficult to refuse.

In the non-WPV subgroup, RC6 (“I am unsure whether my work will be accepted by my superiors”) appeared to be a relatively important bridge item. This may suggest that concerns about recognition, evaluation, and performance acceptance are more closely associated with CCB-related experiences among nurses without WPV exposure. For these nurses, Compulsory Citizenship Behavior may be less related to overt workplace threats and more related to uncertainty about whether their work performance meets supervisory expectations (26, 41, 42).

These findings may have practical implications for nursing management. Managers may consider strengthening role clarification, transparent task allocation, fair evaluation mechanisms, and supportive feedback systems. For nurses exposed to WPV, safe reporting channels, zero-tolerance policies toward workplace violence, and organizational support may be particularly important. Nevertheless, these implications are tentative and should be verified through future longitudinal or experimental research.

### Strengths and limitations

This study possesses significant strengths in terms of methodological innovation and practical application value; however, it also has limitations in design and data that need to be addressed in future research.

### Strengths

First, methodological innovation and rigor. This study is among the first in nursing psychology to apply network analysis models (Ising/EBICglasso) together with simulation-based analytical techniques to examine item-level associations between role clarity and Compulsory Citizenship Behavior (CCB). Compared with traditional regression approaches, network analysis allows visualization and quantification of statistical relationships among individual items and enables exploration of bridge nodes and network pathways. Rather than relying solely on total scores, this approach provides a more detailed description of how specific RC and CCB items are statistically interconnected.

Second, the identification of subgroup-specific network patterns. Through subgroup network analysis based on workplace violence exposure, this study identified differences in the estimated network structures of the two subgroups. RC2

(“I know my job responsibilities”) emerged as a potentially important candidate node across both groups, whereas CCB1 and RC6 appeared to occupy relatively prominent bridging positions in different subgroup networks. These findings may provide useful information for future organizational assessment and intervention research.

Third, the practical relevance of the findings. The simulation-based prediction analysis highlighted several candidate nodes that may warrant further investigation in longitudinal and intervention studies, particularly RC2 and CCB5. Although these findings should not be interpreted as evidence of actual intervention effectiveness, they may help inform the development of more targeted organizational support strategies for junior nurses. The study therefore offers a preliminary framework for identifying potentially important areas of role clarification, supervisory communication, and organizational support in nursing management.

### Study limitations

Several limitations should be acknowledged. First, this study adopted a cross-sectional design, which limits the ability to determine temporal order or causal relationships among role clarity, workplace violence, and Compulsory Citizenship Behavior. Although network analysis can identify complex patterns of statistical associations, it cannot determine whether one item causes changes in another.

Second, all variables were measured using self-report questionnaires at a single time point. Therefore, recall bias, social desirability bias, and common method variance may have influenced the observed associations. Although anonymous participation and voluntary completion were used to reduce response bias, future studies should use longitudinal designs, multi-source data, or objective organizational indicators.

Third, convenience sampling was used to recruit junior nurses from 18 Grade A tertiary hospitals in three provinces of China. Although the sample size was relatively large, the findings may not be generalizable to junior nurses working in other regions, lower-level hospitals, private hospitals, community healthcare institutions, or different healthcare systems.

Fourth, Likert-scale responses were dichotomized for the Ising network model. This approach may have led to information loss, reduced variability, and changes in edge weights, centrality indices, and network structure. Future research should conduct sensitivity analyses using network models suitable for ordinal or continuous data, such as mixed graphical models or Gaussian graphical models.

Fifth, the simulation-based prediction analysis was based on assumptions embedded in the estimated cross-sectional network model. It should not be interpreted as evidence of actual intervention effects. The identified candidate nodes require verification through longitudinal or experimental studies.

Sixth, several potentially important confounding factors were not included, such as leadership style, organizational culture, department type, staffing level, workload, social support, psychological resilience, and career identity. These variables may influence both role clarity and CCB and should be incorporated in future research.

Finally, the estimated network structure may be influenced by measurement characteristics, including item wording, conceptual overlap between scales, and response formats. Therefore, some observed associations may partly reflect measurement-related overlap rather than substantive psychological or organizational processes.

## Conclusion

This study explored the item-level network associations between role clarity and Compulsory Citizenship Behavior among junior nurses and compared the network structures between nurses with and without workplace violence exposure. The findings showed that several role clarity and CCB items occupied relatively important positions in the estimated networks, suggesting that role-related perceptions and involuntary extra-role behaviors may be closely associated among junior nurses. The subgroup analysis indicated that the pattern of associations differed between the WPV-exposed and non-WPV groups. In the WPV-exposed subgroup, CCB-related indicators involving pressure and forced assistance appeared to be more salient, whereas in the non-WPV subgroup, uncertainty about whether one's work would be accepted by superiors emerged as a potentially important bridge item. These findings suggest that WPV exposure may be associated with different patterns of role-related and CCB-related experiences. The simulation-based prediction analysis further suggested that hypothetical changes in selected nodes, particularly those related to clear understanding of job responsibilities, were associated with predicted changes in CCB levels within the estimated network model. However, these results should not be interpreted as evidence of actual intervention effects. Because this study used a cross-sectional self-report design, causal relationships cannot be established. Overall, this study provides preliminary and exploratory evidence regarding the network associations between role clarity and Compulsory Citizenship Behavior among junior nurses. The findings may help identify candidate areas for future organizational assessment and intervention research. Future longitudinal, multi-source, and experimental studies are needed to determine whether role clarity, organizational support, and workplace violence are prospectively associated with changes in Compulsory Citizenship Behavior among junior nurses and whether these factors represent meaningful targets for organizational intervention.

## Data Availability

The original contributions presented in the study are included in the article/supplementary material, further inquiries can be directed to the corresponding author.
